# Performance Evaluation and Oil Displacement Effect of Amphiphilic Polymer Heavy Oil Activator

**DOI:** 10.3390/molecules28135257

**Published:** 2023-07-06

**Authors:** Jiqiang Zhi, Yikun Liu, Jinfeng Chen, Nan Jiang, Dezhu Xu, Lifeng Bo, Guohui Qu

**Affiliations:** 1 School of Petroleum Engineering, Northeast Petroleum University, Daqing 163318, China; zhijiqiang@nepu.edu.cn (J.Z.); cjf030678@163.com (J.C.); sygczrz@stu.nepu.edu.cn (L.B.); quguohui@nepu.edu.cn (G.Q.); 2 Key Laboratory of Enhanced Oil Recovery, Northeast Petroleum University, Ministry of Education, Daqing 163318, China; 3 School of Electrical Engineering & Information, Northeast Petroleum University, Daqing 163318, China; dqjiangnan@nepu.edu.cn

**Keywords:** amphiphilic polymer activator, microscopic remaining oil, nuclear magnetic resonance, laser confocal, CT scanning

## Abstract

A heavy oil activator is an amphiphilic polymer solution that contains hydrophilic and oleophobic groups. It can enhance heavy oil recovery efficiency. This paper studied the changes in the distribution of the remaining oil after activator flooding and the performance of heavy oil’s active agent. Nuclear magnetic resonance spectroscopy, laser confocal microscopy, microscopic visualization, and CT scanning techniques were used to analyze crude oil utilization, and the distribution characteristics of the remaining oil during activator flooding of heavy oil. The results showed that the heavy oil activator solution presented a dense spatial network and good viscosification ability. The activator could reduce the interfacial tension of oil and water, disassemble the heavy components of dispersed heavy oil and reduce the viscosity of heavy oil. The utilization degree of the remaining oil in small and middle pores increased significantly after activator flooding, the remaining oil associated with membranous-like and clusterlike structures was utilized to a high degree, and the decline of light/heavy fraction in heavy oil slowed down. Heavy oil activator improved the swept volume and displacement efficiency of heavy oil, playing a significant role in improving the extent of recovery of heavy oil reservoirs.

## 1. Introduction

**Figure 1:**
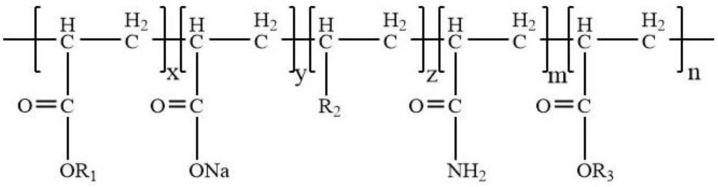
Representation of the structure of the heavy oil activator.

**Figure 2:**
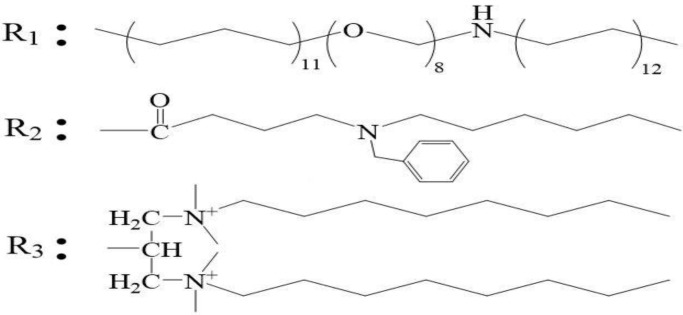
Structure of the functional monomer in the heavy oil activator system.

Heavy oil is characterized by a high viscosity, poor flow capacity, seepage resistance, and low sweep efficiency. It is prone to a pointing phenomenon, which results in a rapid increase in water production, a rapid decline in production, and a low recovery rate. The process of heavy oil activator flooding can be exploited to develop heavy oil reservoirs efficiently. An amphiphilic-polymer heavy oil activator (referred to as “heavy oil activator” for short) is a water-soluble polymer characterized by its high relative molecular weight and interfacial activity. Polyacrylamide functions as the skeleton of the activator, while a regulatory-molecule hydrophilic monomer (Monomer 1, R1), a high-resistance side group (Monomer 2, R2), and gemini amphiphilic functional monomer (Monomer 3, R3) enhance the viscosity, salt resistance, temperature resistance, and solubility of the activator system. These groups also help improve the interfacial activity of the systems. The schematic representation of the activator is shown in Figure 1 and Figure 2. The activator contains hydrophilic and oleophilic groups and is a multifunctional oil displacement agent that can increase the viscosity of the aqueous phase and reduce the viscosity of heavy oil through the processes of disassembly and dispersion. This helps to not only expand the sweep but also improves the efficiency of the oil-washing process, resulting in an increase in the degree of heavy oil recovery realized. At present, research on surfactants mainly focuses on the association, viscosity-increasing effect, emulsification, and oil displacement performance [1,2]. Research on the use of activators in heavy oil reservoirs has rarely been conducted. Therefore, it is necessary to study the distribution law defining micro remaining oil, identify the state of occurrence and distribution characteristics of the remaining oil in different phases of heavy oil under water-flooding conditions and heavy oil activator flooding, determine the use of different types of micro remaining oil, and formulate targeted measures to explore the potential use for the remaining oil. The results present a platform for the development of heavy oil [3,4,5,6].

Researchers worldwide [7,8,9,10,11] have conducted studies to understand the distribution properties of the micro remaining oil in reservoirs. Laser confocal scanning microdetection [12,13,14,15,16,17], nuclear magnetic resonance spectroscopy [18,19,20], CT scanning, and microvisual oil displacement techniques have been used to arrive at results [21,22]. The microscopic remaining oil samples were quantitatively analyzed using nuclear magnetic resonance spectroscopy, laser confocal scanning, CT scanning, and microscopic visualization displacement techniques to understand the state of occurrence and distribution characteristics of the systems. The saturation and morphological changes occurring in different types of remaining oil samples were studied to understand the driving force behind the formation of these systems to provide some theoretical support for the effective development of heavy oil reservoirs.

## 2. Results and Discussion

### 2.1. Performance of Heavy oil Activators

1. Morphological characteristics of heavy oil activator aggregates

**Figure 3:**
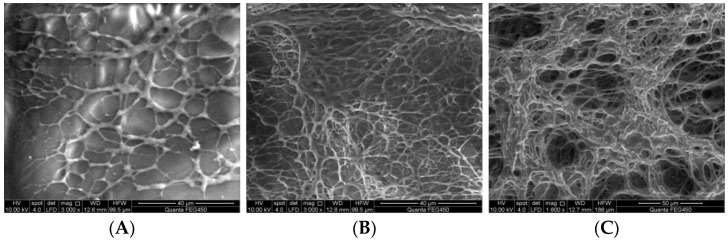
SEM images recorded for the aggregates of activators varying in heavy oil concentrations: (**A**) 600 mg/L; (**B**) 1200 mg/L; (**C**) 1600 mg/L.

Figure 3 presents the morphology of molecular aggregates of the heavy oil activators. The images were recorded using the SEM technique. The analysis of the figure revealed that the intramolecular, intermolecular, and self-cross-linking forces associated with the heavy oil activator units were strong. The activator’s molecular chains intertwined to form aggregates of a certain size, forming a spatial network structure containing many cavities. The density of the network structure increased as the mass concentration of the activator increased. This structure was characterized by a high solubilizing ability for large-space nonpolar nuclei. It could effectively disperse, peel, and carry heavy oil. The structure also presented a strong ability to increase the viscosity of the water phase, and this can result in the expansion of the swept volume during oil flooding.

2. Thickening effect of heavy oil activators

**Figure 4:**
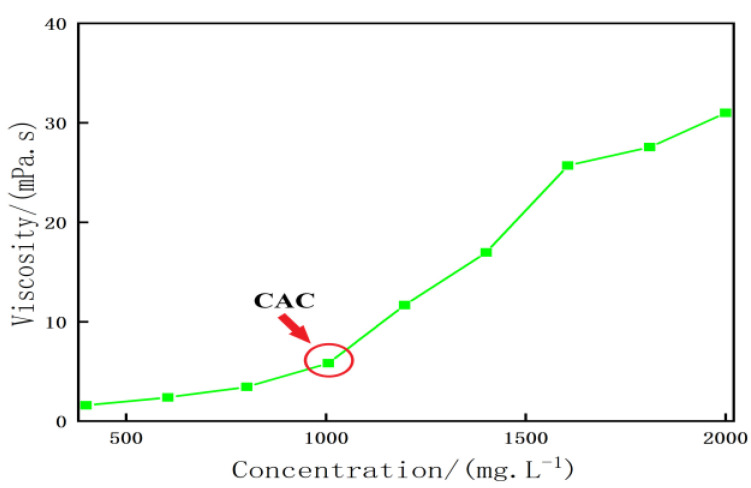
Relationship between the concentration of the activator solution and the viscosity of heavy oil.

Figure 4 presents the relationship between the concentration of the heavy oil activator solution and the viscosity of the solution. The figure shows that the viscosity–concentration curve presented a distinct turning point at 1000 mg/L, corresponding to the critical aggregation concentration (CAC) of the heavy oil activator solution. When the mass concentration of the heavy oil activator exceeds the CAC, macromolecular chains aggregate through hydrophobic association, forming a supramolecular structural–dynamic physical cross-linking network that primarily focuses on intermolecular association [23]. The association between the molecules of the activators was significantly enhanced, resulting in the generation of a unique spatial network. It also resulted in a sharp increase in the viscosity of the activator solution.

3. Interface characteristics of heavy oil activators

**Figure 5:**
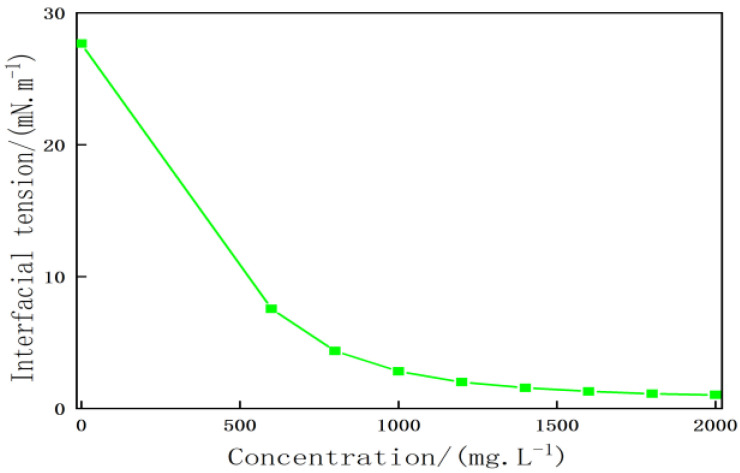
Relationship between the concentration of the activator solution and the interfacial tension of heavy oil.

Figure 5 presents the interfacial tension between the heavy oil activator solution and the heavy oil sample at different concentrations. The analysis of the figure revealed that the oil–water interfacial tension gradually decreased as the concentration of the heavy oil activator solution increased. The initial oil–water interfacial tension corresponding to heavy oil was recorded to be 27.6 mN/m. The oil–water interfacial tension decreased to 1.04 mN/m when 2000 mg/L of the heavy oil activator solution was added to the system. The rate of decrease in the oil–water interfacial tension decreased beyond the heavy oil activator concentration of 1400 mg/L. This can be attributed to the fact that the heavy oil activator molecules linked to a large number of amphiphilic functional monomers with gemini structure units. This can effectively reduce the oil–water interfacial tension, generating a certain level of interfacial activity. The degree of disorder of the activators on the interface increased under the conditions of association between the molecules of the heavy oil activator. Ultralow interfacial tension (10^−3^ mN/m) between the heavy oil activator solution and heavy oil could not be generated.

**Figure 6:**
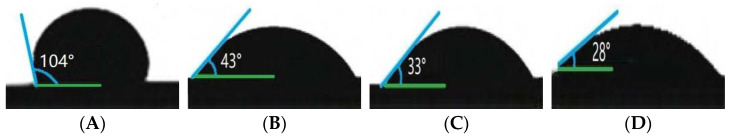
Contact angle corresponding to the activator on the surface of heavy oil of different concentrations: (**A**) 0 mg/L; (**B**) 600 mg/L; (**C**) 1200 mg/L; (**D**) 1600 mg/L.

The contact angles for the activator solutions of different concentrations on the surface of heavy oil are shown in Figure 6. The initial contact angle corresponding to the formation water on the heavy oil surface was recorded to be 104°, and the contact angle corresponding to the 1600 mg/L surfactant solution on the heavy oil surface was reduced to 28°. An increase in the concentration of the heavy oil activator solution resulted in an increase in the spreading ability of the activator solution on the surface of the oil film. The affinity of the activator solution for heavy oil increased under these conditions. The origin of this affinity could be attributed to the strong hydrogen bonding, hydrophobic, electrostatic, π-π stacking, and other forces between the functional monomers in the activator molecular chain and the heavy components in the heavy oil samples.

4. Viscosity reduction effect

**Figure 7:**
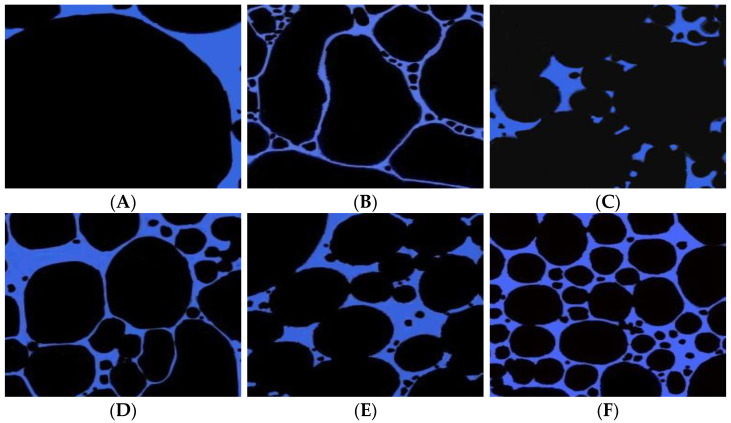
Microscopic image recorded for the heavy oil dispersion system in the presence of activator solutions of different concentrations (100 μm): (**A**) 0 mg/L; (**B**) 600 mg/L; (**C**) 800 mg/L, (**D**) 1000 mg/L; (**E**) 1200 mg/L; (**F**) 1500 mg/L.

The activator solution of different concentrations was mixed with the heavy oil, and the mixture was allowed to stand for 2 h. The microscopic images (laser confocal) of the dispersion system consisting of the surfactant solution and the heavy oil are shown in Figure 7. The particle size of the oil droplets in the dispersion system consisting of the active agent solution and the heavy oil decreased with an increase in the concentration of the active agent. The dispersion effect of the active agent on the heavy oil was enhanced, and the heavy oil was disassembled to the micron level in the dispersed phase. This could be primarily attributed to the fact that the active agent was readily adsorbed at the oil–water interface, and the active agent molecule exhibited a strong affinity for the heavy oil component. The active agent solution could be readily spread on the system, and it could penetrate into the heavy oil component’s aggregation structure. The weak bonding aggregates between asphaltene and gum broke, resulting in the generation of a loose oil–water structure. This resulted in the generation of lateral sliding forces under the lubricating effect of the water film around the hydrophilic group. The heavy oil was dispersed into smaller droplets under these conditions. The disassembling and dispersing action of the active agent solution results in a decrease in the viscosity of the heavy oil system.

**Figure 8:**
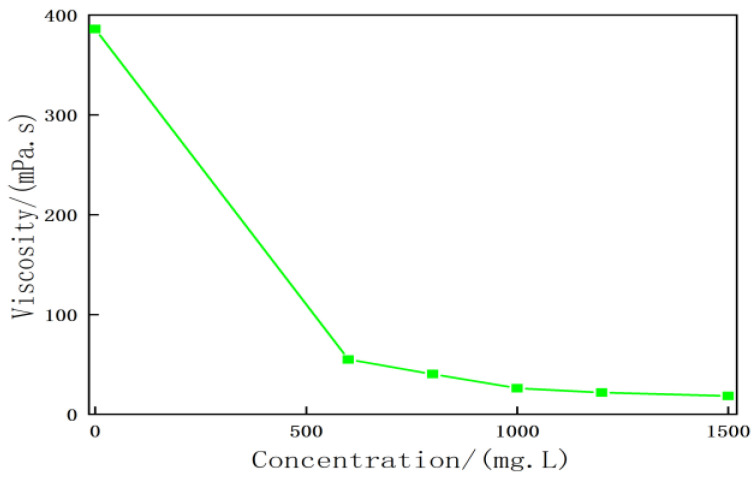
Relationship between the viscosity reduction effect between the activator solution and the heavy oil system.

The heavy oil and activator solution were mixed at an oil-to-water mass ratio of 1:1. As shown in Figure 8, the viscosity reduction effect exerted by the activator solution on heavy oil increased with an increase in the mass concentration of the heavy oil activator system. The viscosity of the heavy oil activator solution–heavy oil dispersion system decreased to 54–18 mPa·s after viscosity reduction when the concentration of the activator solution was in the range of 600–1500 mg/L. The viscosity reduction rate recorded for the heavy oil activator (viscosity of heavy oil: 386 mPa·s at 60 °C) was in the range of 86–95%, both greater than 85%. At a concentration of 1200 mg/L, the viscosity of the activator solution–heavy oil dispersion system was recorded to be 23 mPa·s after viscosity reduction, and the maximum viscosity reduction rate recorded under these conditions was 94%.

### 2.2. Microscopic Oil Displacement Visualization Technology

#### 2.2.1. Microscopic Displacement Effect of Heavy Oil Activators

**Figure 9:**
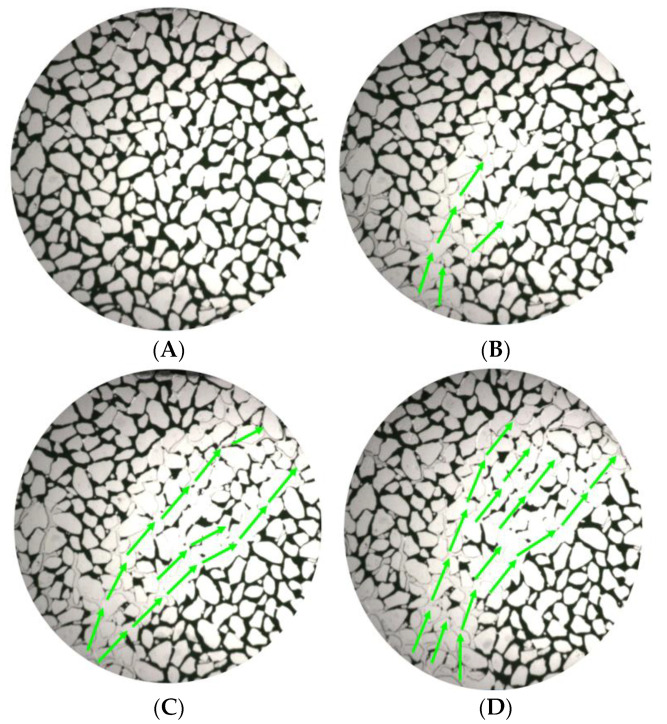
Saturation changes occurring during the microdisplacement water flooding and heavy oil activator flooding stages. (**A**) Initial and (**B**) water flooding process. (**C**) End of water flooding. (**D**) End of heavy oil activator flooding.

The physical simulation of microscopic displacement was conducted to study the mechanism associated with the process of microscopic oil displacement. This was achieved by conducting microscopic oil displacement experiments on photochemical-etching simulation glass models. During the experiment, images were analyzed to quantify the amount of remaining oil in the displacement process [24]. Figure 9 presents an example of a microscopic displacement visualization experiment. This was used to investigate the characteristics of the remaining oil samples and study the displacement effects and microscopic seepage mechanisms associated with water flooding and heavy oil activator flooding.

Table 1 presents the data on the different types of remaining oil. The use of microscopic displacement visualization simulation technology revealed that water flooding predominantly proceeded along the mainstream line area, while the area outside the mainstream line contained a large amount of remaining oil. Due to the high viscosity of heavy oil, the high mobility ratio, and the occurrence of the viscous finger phenomenon, this area was characterized by a low application degree and sweep area. In contrast, heavy oil activator flooding increased the swept area of the flooding system and the displacement pressure and efficiently displaced the columnar-like and clusterlike remaining oil in areas where water flooding was ineffective; the clusterlike remaining oil decreased from 28.18% to 22.60%, and the columnar-like remaining oil decreased from 9.43% to 6.82%. Furthermore, the viscoelastic properties of heavy oil, which resemble those of polymers, and the properties of its active groups make it suitable for displacing remaining oil, presenting membranous-like and corner-shaped geometries; the membranous-like remaining oil decreased from 9.89% to 6.41%, and the corner-shaped remaining oil decreased from 6.76% to 4.57%.

#### 2.2.2. Analysis of the Microdisplacement Mode of the Heavy Oil Activator

**Figure 10:**
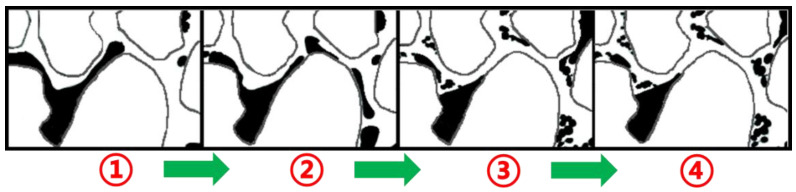
Schematic diagram of the heavy oil activator micro remaining oil displacement mode.

Results from the microscopic displacement experiment revealed that the heavy oil activator was viscoelastic and exhibited the ability to improve the oil–water interface. On the one hand, the high viscosity of the heavy oil activator and the strong interaction between the active groups and heavy oil resulted in the formation of an interfacial film during the displacement of the remaining oil. The shear force acting on the interface during displacement caused the remaining oil to form a tip that could be continuously stretched and fractured. The small oil droplets formed following the fracture of the sample were then transported by the activator, allowing the remaining oil to be utilized. On the other hand, the active groups in the heavy oil activator exhibited excellent interfacial activity, enabling them to reduce the interfacial tension between oil and water, alter the degree of wettability, and break down the heavy oil particles into small droplets. These droplets could be readily separated from the interface and adsorbed onto the oil and water interface, as depicted in Figure 10.

### 2.3. Use of the NMR Technique

**Figure 11:**
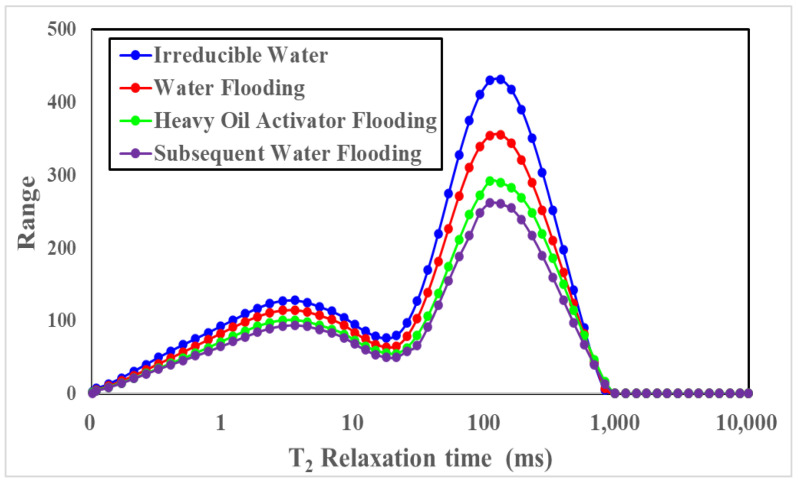
NMR T_2_ spectral profiles recorded at different displacement states.

The data obtained from the NMR experiments were processed to generate the T_2_ spectra of the core at different displacement stages (Figure 11). The T_2_ relaxation time of each pore varied, and those with a relaxation time of less than 10 ms were considered to be small pores. Pores presenting a relaxation time in the range of 10–100 ms were defined as middle pores, while those characterized by a relaxation time greater than 100 ms were considered macropores.

**Figure 12:**
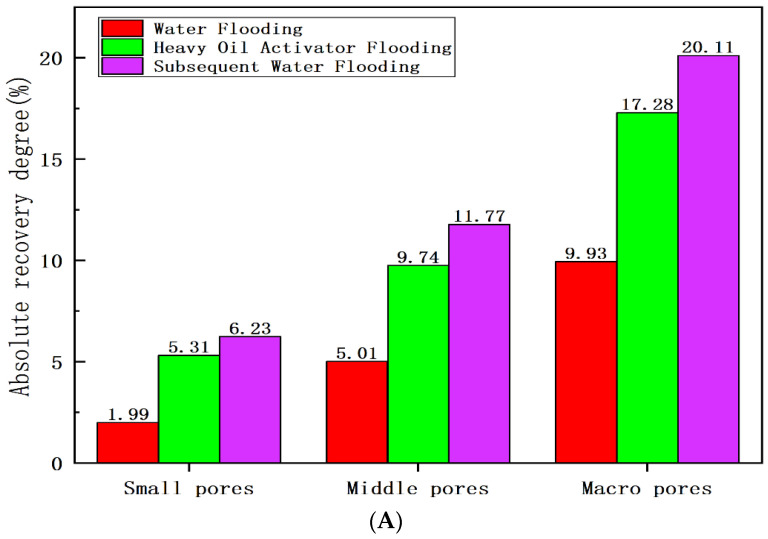

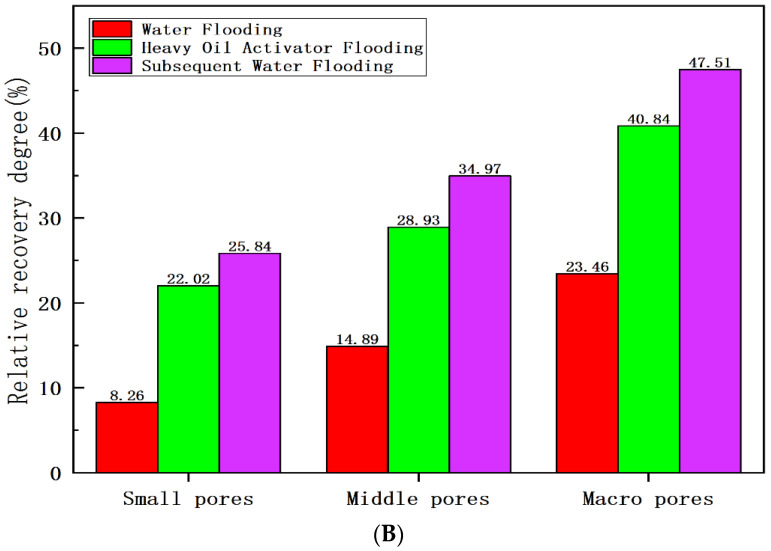
Recovery degree of heavy oil at different displacement stages: (**A**) absolute recovery degree; (**B**) relative recovery degree.

Figure 12, Table 2 and Table 3 present the results obtained for different displacement stages obtained using the NMR technique. The initial oil saturation of the core was 74.17%, and the initial oil saturation of macropores and middle pores was high. The relative recovery degree recorded for the macropores was high (23.46%) after water flooding, and this could be attributed to the high viscosity of heavy oil, a high mobility ratio, and the prominent viscous finger phenomenon. The low development and utilization degree of small and middle pores (middle pores: 14.89%; small pores: 8.26%) resulted in an increase in the degree of retention of the remaining oil; the remaining oil saturation of water flooding was 61.63%. The peak value of the NMR T_2_ spectral curve shifted to the left, and the extent of the remaining oil utilization in small and middle pore spaces increased significantly (28.39% for the middle pore space and 22.02% for the small pore space) following heavy oil activator displacement. The heavy oil activator increased the viscosity of the displacement agent, improved the mobility ratio, expanded the spread volume, and effectively used the remaining oil in small and middle pore spaces. The activator reduced the oil–water interfacial tension, improved the efficiency of the oil-washing process, and reduced the remaining oil content in the swept area. The dissolving and dispersing effect of the crude oil on the heavy oil components reduced the viscosity of the crude oil, increased the fluidity of the system, and helped achieve mobility control. This further expanded the swept volume and oil-washing efficiency, effectively developing the remaining oil in different pore spaces.

### 2.4. Use of the Laser-Scanning Confocal Microscopy Technology

#### 2.4.1. Microscopic Remaining Oil Displacement Effect

**Figure 13:**
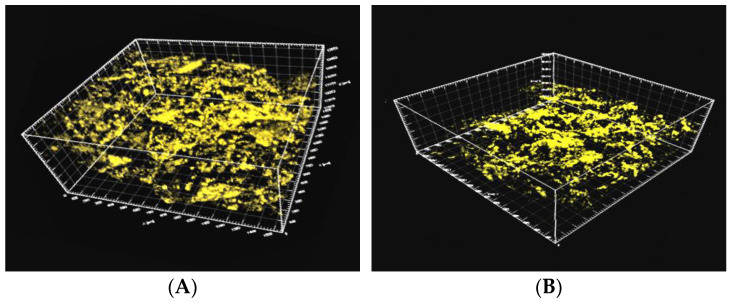
Three-dimensional distribution of the remaining oil studied using the laser confocal microscopy technique at different displacement stages. (**A**) Water flooding; (**B**) heavy oil activator flooding.

We conducted water flooding and heavy oil activator displacement experiments [25,26,27] on natural cores to analyze the oil displacement mechanism of the heavy oil activator and the distribution characteristics of the remaining oil. We then analyzed the distribution characteristics of the remaining oil in the cores using the laser confocal analysis technique to study the core slices. Figure 13 presents the distribution of the remaining oil. The injected water rapidly broke along the direction of the mainstream line during the water flooding stage of heavy oil, resulting in a low degree of utilization for the small and middle pore spaces. It also resulted in the generation of a small sweep area. A large number of clusterlike remaining oil samples was not utilized, and these were primarily distributed in the pores in the form of oil beads, clumps, and clusters. A considerable amount of the film formed from the remaining oil was absorbed on the surface of the membranelike material, and this could be attributed to the presence of asphaltene, colloidal compounds, and other components in the heavy oil. A significant amount of the clusterlike remaining oil was driven out from the system after heavy oil activator flooding, and the content of the membranelike remaining oil was significantly reduced under these conditions. We compared the distribution characteristics of the remaining oil (water flooding) and heavy oil (activator flooding). We found that the functional groups in heavy oil activators could expand the swept volume and improve the washing efficiency, effectively displacing free and bound remaining oils in the core.

**Figure 14:**
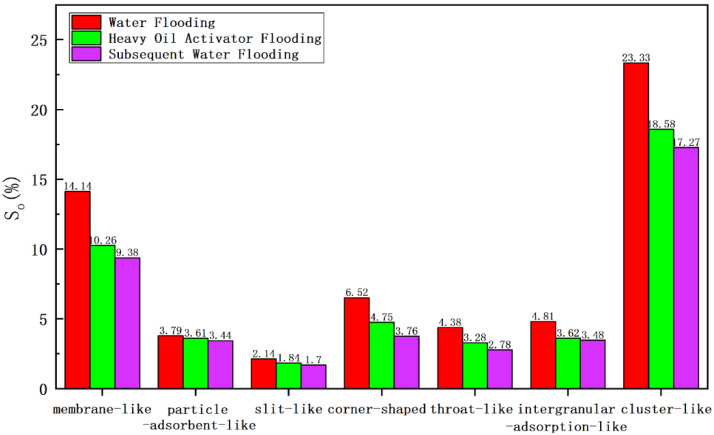
Comparison of the data obtained for the remaining oil using the laser confocal microscopy technique.

We prepared sample slices after core water flooding and heavy oil activation agent flooding and used the laser confocal technology to identify the oil–water boundary. We also used this technology to construct a three-dimensional distribution map for the remaining oil. The relative contents of each type of microscopic remaining oil samples were recorded for different displacement stages (Table 4 and Figure 14).

The recovery degree was low (16.81%), and the remaining oil content was high (49.25%) during the water flooding stage (Table 4). Clusterlike and membranelike remaining oil samples accounting for 63.39% of the remaining oil content, were present in the system. The clusterlike and membranelike remaining oil samples exerted the best utilization effect during heavy oil activator flooding, decreasing the remaining oil saturation level (from 23.33% and 14.14% to 18.58% and 10.26%, respectively). The degree of utilization followed the order of corner-shaped remaining oil > intergranular-adsorption-like remaining oil > throatlike remaining oil. The remaining oil saturation level decreased from 6.52%, 4.81%, and 4.38% to 4.75%, 3.62%, and 3.28%, respectively.

Figure 14 compares the contents of different types of remaining oil samples obtained after water flooding and heavy oil activator flooding. The results revealed that clusterlike and membranelike remaining oil samples were used to the maximum extent after activator flooding. This can be explained as follows:

Firstly, during heavy oil–water flooding, the high water flow ratio and the prominent viscous finger phenomenon result in the generation of a large number of clusterlike remaining oil samples in the mainstream line area that cannot be displaced by water flooding. However, the association between heavy oil activator molecules results in the formation of a spatial network that increases the viscosity of the water phase, reduces the water flow ratio for oil, improves the displacement pressure, expands the swept volume, and floods the clusterlike remaining oil in the pores of the heavy oil activator.

Secondly, the content of the membranelike remaining oil is high in the water flooding stage, and this can be attributed to the weak adhesion ability of water and the small shear force. Therefore, the ability of the membranelike remaining oil to get attached to the pore surface is low. However, the use of heavy oil activators can help reduce the interfacial tension between oil and water, change the wettability of the core, and reduce the degree of adhesion at the pore wall. This results in the thinning and detachment of the membranelike remaining oil. Additionally, the heavy oil activator exerts a disassembling and dispersion effect on heavy oil components. This promotes the disassembly of heavy oil to form a micron-level dispersed phase. It also helps improve the oil-washing efficiency and results in the utilization of the membranelike remaining oil.

The utilization degree of the corner-shaped, intergranular-adsorption-like, and throatlike remaining oil samples was high, and this could be potentially explained by the following factors: corner-shaped remaining oil is closed at one end, resulting in the formation of a blind end that cannot be effectively displaced during water flooding. However, a heavy oil activator with its viscoelasticity can be used to “drag” and “peel” the remaining oil. The intergranular-adsorption-like remaining oil is primarily distributed in areas with high mud and clay content. The heavy oil activator reduces the interfacial tension between oil and water, and this promotes the usage of some of the remaining oil adsorbed between grains. The formation of throatlike remaining oil can be attributed to the high viscosity of heavy oil present at both ends of the throat and the small difference in the displacement pressure under water-flooding conditions. This arrests the flow of the remaining oil. The unique interfacial activity of heavy oil activators can produce the effect of disassembly. It can help the viscosity reduction, aggregation, and resistance increase, which can eventually help increase the fluidity of heavy oil on both sides of the throat and effectively displace the remaining oil in the throat.

Particle-adsorbent-like and slitlike remaining oil samples fell under the category of the bound remaining oil, and these exhibited poor displacement effects. These were characterized by a relatively low degree of utilization.

#### 2.4.2. Microscopic Remaining Oil Displacement Effect of Different Components

We conducted water flooding and heavy oil activator flooding experiments on natural core samples to investigate the microscopic distribution of the remaining oil in different components of heavy oil. Additionally, we performed experiments on the light and heavy components of the microscopic remaining oil. The fluorescence properties (color and intensity) of crude oil components varied depending on the nature of the components. The composition of crude oil can be determined by analyzing the fluorescence color of rock samples excited using ultraviolet light. We identified the presence of asphaltene, gum, aromatics, and saturated hydrocarbons by analyzing the confocal laser signals and by generating light/heavy oil distribution maps. We observed that the heavy components of heavy oil consisted primarily of asphaltene and colloid, while the light components were primarily the residual components.

**Figure 15:**
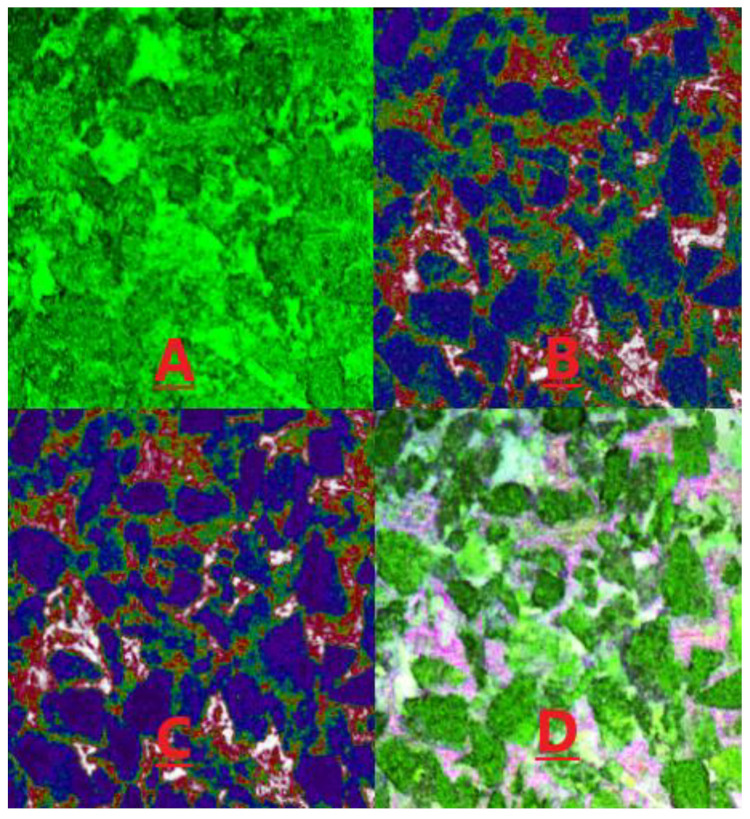
Distribution of light/heavy components under water-flooding conditions. (**A**) the reflected light signal; (**B**) the fluorescence signal corresponding to the light crude oil; (**C**) the fluorescence signal of heavy crude oil; (**D**) composite image of the previous three figures.

**Figure 16:**
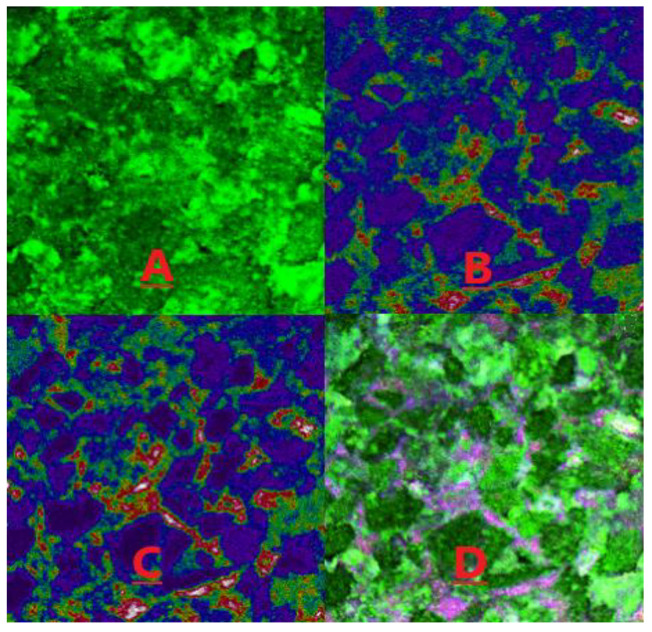
Distribution of the light/heavy components of the microscopic remaining oil under the conditions of heavy oil activator flooding. (**A**) the reflected light signal; (**B**) the fluorescence signal corresponding to the light crude oil; (**C**) the fluorescence signal of heavy crude oil; (**D**) composite image of the previous three figures.

Figure 15 and Figure 16 present the distribution of the light and heavy components of the remaining oil under water flooding and heavy oil activator flooding conditions. In these figures, green represents the water phase, red represents crude oil, white represents the part with a high crude oil content, and purple represents minerals. Figure 15A presents the reflected light signal, which reflects the surface morphology of the minerals. Figure 15B presents the fluorescence signal corresponding to the light crude oil, which reflects the distribution state of the light crude oil. Figure 15C presents the fluorescence signal of heavy crude oil, which reflects the distribution state of heavy crude oil. Figure 15D is a composite image of the previous three figures, and it can directly reflect the distribution of the remaining oil on the mineral surface.

Figure 15 and Figure 16 demonstrate that the water flooding of heavy oil resulted in a poor displacement, leaving a significant amount of remaining oil in the pores of rocks. This can be attributed to the large viscosity difference and other factors. Heavy oil activator flooding improved the mobility ratio through the viscosification effect of the activator system, expanding the swept volume and effectively utilizing the remaining oil that was not affected by water flooding. Additionally, the activator system disassembled and dispersed heavy oil components (asphaltene and gum). This resulted in increased usage, albeit under conditions of a reduced distribution range. We further analyzed the reflected light signal using software to determine the proportion of light/heavy components in the core pores, obtain the saturation and recovery degrees of light/heavy components in the different crude oil samples, and calculate the light/heavy component ratios for the remaining oil (Table 5).

Results obtained using the laser confocal fluorescence detection technique revealed that for the core saturated oil, the content of the light component (residual component) of crude oil was 1.48 times higher than the content of the heavy component (asphaltene and gum). However, the light/heavy ratio was reduced by 1.20 times for the case of water flooding, and this could be attributed to the high viscosity of the heavy components and the strong adhesion force exerted by the heavy components (such as asphaltene and gum) which adhered to the pore wall. The wall wettability was oil-wet, causing a large number of heavy components to get adsorbed on the rock and mineral particle’s surface. This resulted in a low degree of usage of heavy components during water flooding and a high recovery degree of light components.

In contrast, heavy oil activator flooding reduced the light/heavy ratio by 1.12 times, and a low rate of decrease was recorded under these conditions. The viscosification effect of the activator solution increased the swept volume, facilitating the use of the remaining light components in the swept area. Additionally, the activator solution exhibited a good interfacial activity, making the adsorption of the particles on the oil–water interface easy. This increased the affinity between the activator molecule and the heavy oil component, enabling the activator solution to spread and penetrate the aggregated heavy oil component. This resulted in the partial disassembly of the weak binding aggregates formed from asphaltene and gum. This resulted in the generation of a loose oil–water interface structure. A lateral sliding force was generated under the lubrication action of the water film around the hydrophilic groups, and a weak stirring action was recorded under these conditions. As a result, the heavy components were dispersed into small oil droplets, resulting in a significant increase in the recovery degree of the heavy components. The recovery degree for heavy components increased from 3.22 to 9.10% after heavy oil activator flooding.

### 2.5. Results Obtained Using the CT Scanning Technology

#### 2.5.1. Microscopic Remaining Oil Displacement Effect: The 2D CT Scanning Plane

**Figure 17:**
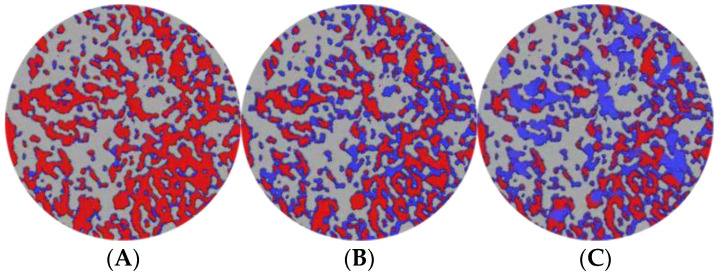
Distribution maps generated at different displacement stages for the remaining oil samples using the CT scanning technique in the 2D plane. (**A**) Initial; (**B**) water flooding; (**C**) heavy oil activator flooding.

Tomography of the cores was recorded at various displacement stages, including initial, water flooding, and heavy oil activator flooding, and 500 sections were scanned for each displacement stage. The scanned data were reconstructed, and the images were optimized by adjusting the threshold brightness parameter, removing noise, and improving the extent of the beam hardening realized. The resulting maps generated for the remaining oil samples were analyzed. The maps were generated based on the two-dimensional CT planes corresponding to different displacement stages (Figure 17). Red represents the oil phase, blue represents the water phase, and gray represents the rock particles.

In the initial state of the core, the remaining microscopic oil primarily existed as a continuous phase (as clusterlike and multiporous forms of the remaining oil). During the water flooding stage, the crude oil was segmented under conditions of water phase displacement, and this resulted in an increase in the proportion of discontinuous remaining oil (columnlike, dropletlike, and membranous-like oil). However, the prevalence of the fingering phenomenon could be attributed to the high viscosity of heavy oil and a high content of the continuous remaining oil was recorded under these conditions.

The spread area expanded due to the viscosification effect of the activator during heavy oil activator flooding, and part of the continuous phase was displaced under these conditions. Additionally, the disassembly and dispersion effect of the activator, along with the reduction in interfacial tension, resulted in a significant reduction in the remaining oil content and the effective use of some of the drip and columnar remaining oil.

#### 2.5.2. Microscopic Remaining Oil Displacement Effect: The 3D CT Scanning Plane

**Figure 18:**
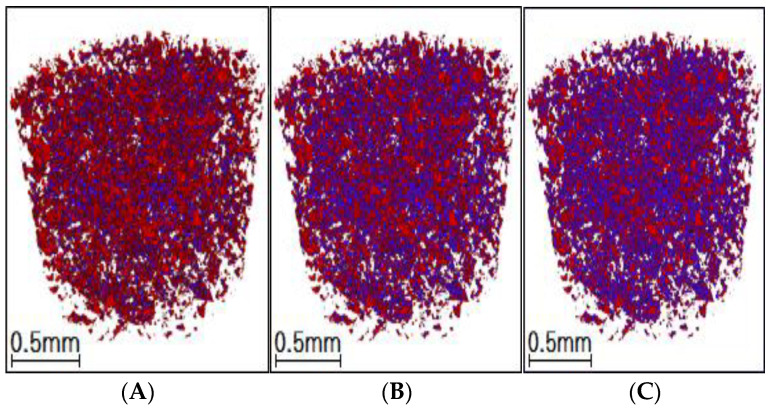
Distribution map generated from 3D CT scans of remaining oil. (**A**) Initial; (**B**) water flooding; (**C**) heavy oil activator flooding.

**Figure 19:**
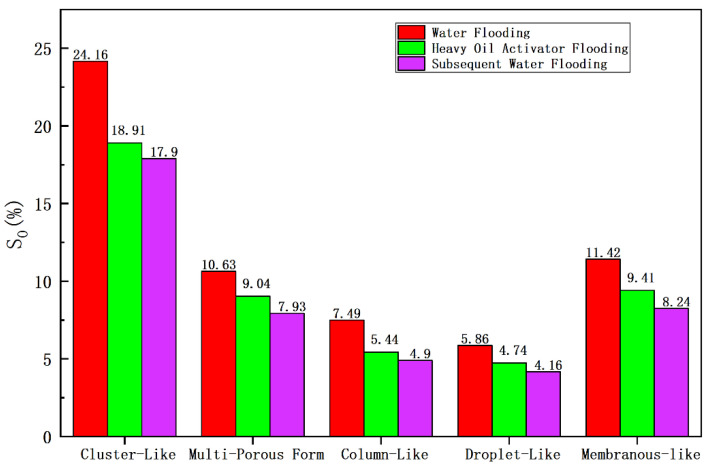
Comparison of the contents of different types of remaining oil samples subjected to CT scan.

A 3D pore network model (Figure 18; the red area represents the oil phase, and the blue area represents the water phase) was constructed using the CT scanning data obtained for cores at different displacement stages. The distribution morphology of the microscopic remaining oil was studied and analyzed, and the results obtained from the quantitative characterization are shown in Table 6 and Figure 19.

The constructed 3D distribution diagram for the remaining oil revealed that the oil phase corresponding to the saturated oil appeared as a continuous phase, and clusterlike and multiporous remaining oil products were usually formed under these conditions. The degree of saturation was recorded to be 42.37%. The oil phase was displaced, scoured, divided, and dispersed during the water flooding stage, resulting in the formation of a large number of columnlike, dropletlike, and membranous-like remaining oil particles. However, due to the heterogeneity of the core, poor oil–water viscosity, and fingering phenomenon, the affected area of water flooding was limited. Clusterlike and multiporous remaining oil was predominantly present after water flooding, and the degree of saturation of the remaining oil was calculated to be 34.79%. Large pores and pore-throat intersections were primarily present in the unswept area. The area affected by heavy oil activator flooding increased under the viscosification effect of the activator. The clusterlike and multiporous form of the continuous phase was effectively utilized, resulting in a drop in the saturation extent of the remaining oil (27.95%). Furthermore, the reduction in interfacial tension and the disassembly and dispersion effect of the activator promoted the effective utilization of the membranelike and columnlike remaining oil. The degree of saturation decreased from 11.42% and 7.49% to 9.41% and 5.44%, respectively, after water flooding.

## 3. Experimental

### 3.1. Materials and Methods

Reagent: the heavy oil activator (relative molecular weight: 600 × 10^4^) was obtained from Beijing Baxter Tech Energy Engineering Technology Co., Ltd., (Beijing, China), and the MnCl_2_ solution (10,000 mg/L, concentration of Mn ions) was obtained from Modern Oriental Technology Co., Ltd., (Beijing, China).

Experimental oil: The formation crude found in common heavy oil reservoirs in Liaohe Oilfield (viscosity: 386 mPa·s) was used as the experimental oil.

Experimental water: the reinjection formation water at the Liaohe oilfield (salinity: 881.3 mg/L).

Experimental core: the common heavy oil reservoir in the Liaohe oilfield was used as the natural core (diameter: 2.5 cm). The core was dry and devoid of cracks.

Experimental instruments: HW-4A thermostat, Hai ‘an Huada Petroleum Instrument Co., Ltd., Nantong, China; TEXAS-500C rotary drop interfacial tensiometer, Beijing Zhongyicexin Co., Ltd., Beijing, China; Micro-displacement Test Platform, Yangzhou Huabao Petroleum Instrument Co., Ltd., Yangzhou, China; SIAS Image Analysis Software, Institute of Image Information, Sichuan University, Chengdu, China; SteREO Discovery V12 microscopes, Beijing Chuangcheng Zhijia Technology Co., Ltd., Beijing, China. MicroMR12 NMR instrument, Suzhou Niumai Analytical Instrument Co., Ltd., Suzhou, China; laser-scanning confocal microscope (LSCM, model LEICA SP5II), Leica Instrument Co., Wetzlar, Germany; Micro XCT-400 CT machine with CT scan image data-processing software Avizo 8.0 were used in this experiment, Xradia Inc., Pleasanton, CA, USA; OLYMPUS BX41 type optical microscope, Olympus Corporation, Tokyo, Japan; Attension Theta Flex contact angle meter, Biolin Scientific corporation, Goteborg, Sweden; QuantaFEG 450 type environmental scanning electron microscope (SEM), FEI Company, Hillsboro, OR, USA; electronic balance (BS124 type), Sartorius Corporation, GER; microsimulation glass etching model, homemade; OLYMPUS BX41 type optical microscope, Olympus Corporation, Tokyo, Japan; micropump, Hai ‘an Petroleum Scientific Research Instrument Co., Ltd., Nantong, China.

### 3.2. Methods

#### 3.2.1. Performance of the Heavy Oil Activator Solution

1. Preparation of the heavy oil activator solution

The mother liquor (5000 mg/L, heavy oil activator solution) was initially prepared, and the prepared liquor was aged and allowed to stand at room temperature for 12–24 h. Following this, the heavy oil activator solution was diluted to different target concentrations, and a Waring agitator was used to stir the solutions (speed: 3000 r/min; simulating the shear of heavy oil activator solution near the well) for 20 s at 60 °C. The solution could be used post shearing and defoaming.

2. Characterization of the aggregates formed from the heavy oil activator solution

The heavy oil activator solutions varying in their concentrations were dropped onto clean silicon surfaces and frozen in liquid nitrogen. The frozen samples were dried in a freeze-dryer. The dried samples were gilded for testing. The morphology of the aggregates was studied using the scanning electron microscopy (SEM) technique.

3. Viscosity of the heavy oil activator

Heavy oil activator solutions differing in concentrations were allowed to stand at 60 °C for 1 h. The samples were stirred for 5 min (400 r/min), and the RS600 high-temperature and high-pressure rheometer was used for the sample analysis. The shear rate was maintained at 7.34 s^−1^ during the viscosity test.

4. Interfacial tension of the heavy oil activator

The rotary drop interfacial tensiometer was used to measure the interfacial tension between the heavy oil activator solution and the heavy oil samples. The solutions differed in their concentration, and the experiments were conducted at 60 °C.

5. Measurement of contact angle for heavy oil activator

The heavy oil was evenly coated on the surface of the slide, and the heavy oil activator solution was dripped onto the surface of the heavy oil film. The contact angle was recorded using the contact-angle-measuring instrument.

6. Determination of dispersion system corresponding to heavy oil activator

The activator solution with different concentrations was prepared, and the heavy oil was mixed and stirred for 10 min at a constant temperature of 60 °C. The rotation speed was 400 r/min. The mixed solution was observed using a laser confocal microscope, and the particle size, particle distribution, and dispersion state of the droplets in the dispersion system were measured.

7. Viscosity reduction performance of heavy oil activator

The heavy oil activator solutions of different target concentrations were prepared, and the heavy oil sample and the activator solution were mixed at the oil/water mass ratio of 1/1. The prepared solution was allowed to stand for 1 h at 60 °C, and the samples were stirred continuously for 10 min (400 r/min). The RS600 high-temperature and high-pressure rheometer was used to determine the viscosity of the sample (shear rate: 7.34 s^−1^).

#### 3.2.2. Microscopic Residual Distribution Characteristics of the Heavy Oil Activator

Nuclear magnetic resonance, laser confocal microscopy, microscopic visualization, CT scanning, and laboratory displacement techniques were used to analyze the crude oil recovery degree, the distribution characteristics of the remaining oil, and the displacement effects of the heavy oil and heavy oil components observed during the activator flooding of the heavy oil. The goal was to study the changes in the distribution of the remaining oil after activator flooding.

$$\mathrm{Recovery}\;\mathrm{degree}=\frac{Cumulative\;displacement\;oil\;production}{Total\;oil\;reserves\;inpores}\times 100\%$$

$$\mathrm{Remaining}\;\mathrm{oil}\;\mathrm{saturation}=\frac{The\;volume\;of\;remaining\;oil\;in\;the\;pores\;at\;the\;end\;of\;displacement}{Total\;pore\;volume}\times 100\%$$

$$\mathrm{Oil saturation}=\frac{Volume\;of\;oil\;in\;pores}{Total\;pore\;volume}\times 100\%$$

$$\mathrm{Ratio}\;\mathrm{of}\;\mathrm{light}/\mathrm{heavy}\;\mathrm{components}=\frac{Volume\;of\;light\;component\;crude\;oil}{Volume\;of\;heavy\;component\;crude\;oil}$$

1. Study of the microscopic displacement property of the sample using the visual oil displacement technology (use of the microscopic lithographic glass model)

**Figure 20:**
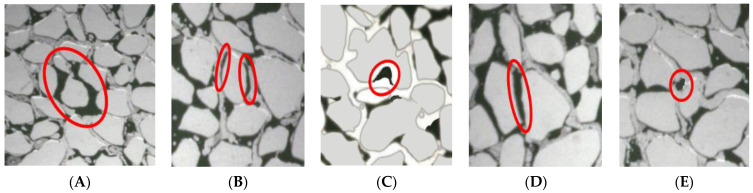
Schematic diagram presenting the distribution morphology of the remaining oil. Schematic representation of the (**A**) clusterlike; (**B**) membranous-like; (**C**) corner-shaped; (**D**) columnar-like; (**E**) dropletlike samples.

The use of the microvisual oil displacement system involves the use of high-speed cameras to capture dynamic images and conduct microscopic displacement experiments. The system controls the displacement speed under the displacement mode of the micropump to study the dynamic migration of oil during the oil displacement process. The distribution characteristics and occurrence ratio were studied by analyzing the state of occurrence of the remaining oil samples at different displacement stages. The experimental steps were as follows:

➀ The crude oil and displacement fluid were filtered, and the microscopic model was vacuumed using a miniature vacuum pump.
➁ Saturated formation water was pumped at the rate of 0.03 mL/min using a micro-pump. The temperature was maintained at 60 °C for 2 h.
➂ Saturated heavy oil was pumped at the speed of 0.03 mL/min using a micropump. The temperature was maintained at 60 °C for 2 h.
➃ We simulated the water flooding process at a constant displacement rate of 0.03 mL/min to drive the oil sample at a steady pace. We studied the microscopic seepage process using the microscopic model. The process was studied using a microscope under water-flooding conditions. The dynamic images of the displacement process were analyzed simultaneously using a high-speed photography system. The process was halted once the moisture content had reached 98%.
➄ We injected the activator solution (0.3 pv) at a constant speed of 0.03 mL/min for the oil flooding to replicate the heavy oil activator flooding process. We studied the microscopic seepage process using the microscopic model (under a microscope) during the heavy oil activator flooding process and recorded the dynamic images of the displacement process using a digital camera.
➅ We studied the microscopic seepage process during the process of water flooding to simulate the subsequent water flooding process. The displacement rate was maintained at 0.03 mL/min, and the process was studied using a microscope. We also recorded the dynamic images of the displacement process using a digital camera. The displacement process was stopped when the moisture content reached 98%.
➆ The image analysis process included both overall and local analysis. These processes helped study the distribution of the remaining oil in the systems. The remaining oil system could be classified into five types based on the pore structure and oil distribution properties of the samples: clusterlike, membranous-like, corner-shaped, columnar-like, and dropletlike (Figure 20).

2. Use of the nuclear magnetic resonance (NMR) spectroscopy technique for sample analysis

An NMR experiment was simultaneously conducted during the displacement experiment. A natural core (diameter: 2.5 cm; length: 10 cm) characterized by a permeability of 1405 mD (gas measurement) was selected for the indoor core displacement experiment. The crude oil exhibited a viscosity of 386 mPa·s at 60 °C. The heavy oil activator solution was prepared using the formation water prepared using heavy water. The resonance frequency of the nuclear magnetic resonance imager was set to 200 MHz. The experimental steps are as follows:

➀ The core was pumped and then saturated with water. Following this, the NMR spectroscopy technique was used to study the core. Subsequently, the core was dried at a temperature of 110 °C, and this was followed by another vacuum-pumping step, during which the heavy-water-treated formation water was pumped into the core.
➁ The core was saturated with oil, and the displacement rate was maintained at 0.1 mL/min under these conditions. The saturated state of the core was scanned using the dynamic NMR technique.
➂ Water flooding was performed on the core sample, and the displacement rate was maintained at 0.1 mL/min. The water content was 98%. The dynamic NMR spectroscopy technique was used to analyze the core during water flooding.
➃ The heavy oil activator was injected into the core at the displacement rate of 0.1 mL/min after water flooding. The heavy oil activator solution (0.3 pv) was injected into the core for dynamic NMR scanning.
➄ A subsequent water flooding step was carried out, and oil could not be produced at the production end after heavy oil activator flooding. The dynamic NMR spectroscopy technique was used to scan the core during displacement.

3. Use of the laser confocal microscopy technique

The laser confocal scanning microscopy technology is a microscopy-based analysis technique that combines high-speed laser scanning, wet microtechnology, and image processing technologies. It is used to study the microstructure of rock reservoirs, and high-definition images of the internal layers and structures can be obtained by freezing core slices. The microscopic mechanism of heavy oil displacement, the distribution characteristics of the remaining oil, and the application prospects of heavy and heavy components can be studied using this technology. The experimental steps were as follows:

➀ The natural core was saturated at the temperature of 60 °C.
➁ The core was water-flooded (water content: 98%), and sample slices were prepared. The saturation level of the remaining oil, the oil/water area, and the contents of different types of remaining oil were analyzed using the laser confocal and computer image processing technologies.
➂ The heavy oil activator was injected into the core, and the activator solution (0.3 pv) was injected into the system. The saturation level of the remaining oil, the oil/water area, and the contents of different types of remaining oil were determined using laser confocal technology.
➃ A subsequent water flooding step was carried out to displace the unproduced oil at the production end, and the microscopic remaining oil in the core was studied using laser confocal technology.

**Figure 21:**
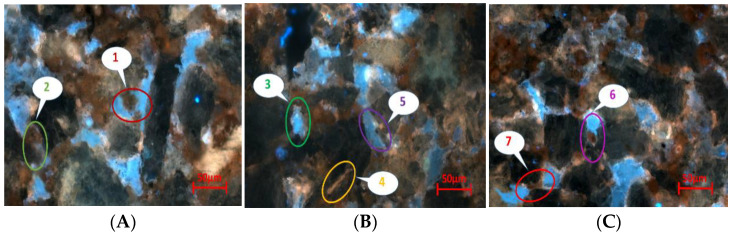
Distribution types of remaining oil studied by analyzing the scanning image recorded using a laser confocal microscope: (**A**) free remaining oil; (**B**) bound remaining oil; (**C**) semibound remaining oil: 1. clusterlike, 2. intergranular-adsorption-like, 3. membranelike, 4. slitlike, 5. particle-adsorbent-like, 6. corner-shaped, 7. throatlike.

The types of remaining oil and the distribution characteristics of the samples were analyzed by analyzing the images recorded (Figure 21). The remaining oil samples were divided into three categories.

Free remaining oil: It can be further classified into two subtypes, clusterlike and intergranular-adsorption-like remaining oil. This type of oil is predominantly distributed at a distance from the pore wall surface. Clusterlike remaining oil is present in the pores in the form of oil beads, clumps, and clusters. Intergranular-adsorption-like remaining oil, on the other hand, is primarily found in areas with a high content of clay mineral components or intergranular mud impurities.

The second type of remaining oil is referred to as bound remaining oil, which includes membranelike, slitlike, and particle-adsorbent-like remaining oil. This type of oil is primarily adsorbed on the surface of the pore wall. Membranelike remaining oil presents itself in the form of a film that adheres to the surface of rock and mineral particles. Slitlike remaining oil is primarily found in narrow gaps that are thin and long (less than 0.01 mm in length). Particle-adsorbent-like remaining oil is primarily distributed on the surface of rock mineral particles through adsorption.

The third type of remaining oil is known as semibound remaining oil, and it consists of the corner-shaped and throatlike remaining oil. This type of oil is primarily found outside the bound remaining oil and is relatively distant from the pore wall surface. The corner-shaped remaining oil is commonly present in complex pore spaces, occupying the corners of the pores where one side is located in the pore depression while the other side is connected to the external space. Throatlike remaining oil is usually located in small throats that are connected by pores due to the capillary effect.

4. CT scanning technique

Microcomputed tomography (Micro-CT) was used as the CT scanning technology [28]. After the displacement experiment, the core was subjected to X-ray scanning to obtain X-ray attenuation coefficient data within the scanning area for each substance. Subsequently, a three-dimensional reconstruction of the data volume was carried out, and the image was optimized by adjusting threshold brightness, removing noise, and improving the beam-hardening properties. Professional software was then used for the image segmentation and pore network model establishment. The distribution of the oil phase, water phase, and particle phase in the core was studied by extracting each of the phase fluids and rock skeleton particles from the image and model. The Euler number, contact ratio, and shape factor of the oil cluster were used to classify the microscopic remaining oil. The position of the core holder remained unchanged in the CT scanner during the entire experimental displacement process, and this enabled in situ scanning. The experimental procedure is as follows:

➀ Preparation of core: A natural core from a heavy oil reservoir was selected and dried at 110 °C to prepare the core for experiments. The core’s basic physical parameters, including permeability and porosity, were studied before vacuuming. The core was first saturated with water and then with oil. The CT scanning method was used to analyze the core in the initial, bound water state, and the scanning data volume was obtained.
➁ Water flooding was carried out at the displacement rate of 0.1 mL/min until the water content reached 98%. The CT scanning method was used to obtain the scanning data volume.
➂ Heavy oil activator flooding was realized, and the activator (0.3 pv) was injected into the core. The CT scanning method was used for the analysis, and the scanning data volume was obtained.
➃ The core was scanned using the CT technique after water flooding to obtain the scanning data volume. Oil was not produced at the production end, indicating that the process of water flooding could effectively displace oil.
➄ Description of the microscopic remaining oil in the core: the scanning data were processed and analyzed, and a three-dimensional reconstruction was realized.

The data volume acquired using the CT scanning technique was analyzed and processed to generate a scanning image. A further reconstruction yielded a 3D map presenting the distribution of the remaining oil. The microscopic remaining oil was classified into five types based on factors such as the location of occurrence, oil–water contact, and flow morphology. These could be classified into the categories of clusterlike, multiporous, columnar-like, dropletlike, and membranous-like systems, as shown in Table 7. The classification was based on the contact ratio of oil clusters, Euler number, and shape factor [29,30].

## 4. Conclusions

The introduction of a functional monomer on the polyacrylamide skeleton in an amphiphilic polymer heavy oil activator increased the viscosity of the displacement agent system, improved the mobility ratio, and expanded the swept volume. The interfacial tension between oil and water was reduced, and the efficiency of the oil-washing process was improved simultaneously. The analysis of the displacement effect exerted by the microscopic remaining oil in the water flooding and heavy oil activator flooding stages revealed that heavy oil presented a low application degree and was characterized by a small, sweet area. This could be attributed to the high viscosity of the materials, high degree of fingering, and poor fluidity of the heavy components. However, heavy oil activator flooding effectively utilized different types of remaining oil in the pores by expanding the sweet volume and improving the efficiency of the oil-washing process. The research findings are summarized below.

1. A dense spatial network structure was formed when a heavy oil activator reached a certain concentration. The viscosity of the activator solution sharply increased when the mass concentration was higher than the CAC (1000 mg/L). The activator solution exhibited a good interfacial activity, which significantly reduced the oil–water interfacial tension. The adsorption–permeation–disintegration–dispersion effect on the heavy oil interface resulted in a reduction in the viscosity of heavy oil (maximum reduction achieved: 94%) when the oil/water mass ratio was 1:1, and the activator solution concentration was 1200 mg/L.
2. The heavy oil activator exhibited viscoelastic characteristics. A strong interaction between the active groups and heavy oil was recorded, and this promoted the formation of an interface film that produced an additional shear effect. This allowed for the effective utilization of membranous-like and corner-shaped remaining oil through a “dragging” mechanism. This resulted in a decrease in the saturation level of the remaining oil from 9.89% (membranous-like) and 6.76% (corner-shaped) to 6.41% and 4.57%, respectively, after water flooding. Furthermore, the activator could increase the viscosity and displacement pressure, enabling the utilization of clusterlike and columnar-like remaining oil. Consequently, the saturation level of the remaining oil decreased from 28.18% (clusterlike) and 9.43% (columnar-like) to 22.60% and 6.82%, respectively, under these conditions.
3. The NMR results indicated that heavy oil activator flooding significantly impacted the utilization of small and middle pore spaces. Specifically, the recovery degree of middle pores increased from 14.89% to 28.39%, which indicated that a large amount of heavy oil was displaced and recovered from the middle pore spaces after activator flooding. Similarly, the recovery degree of small pores increased from 8.26% to 22.02%, which suggested that the activator was able to effectively mobilize and recover heavy oil from the small pore spaces. These results demonstrate that heavy oil activator flooding can improve the degree of utilization and recovery of oil from previously inaccessible pore spaces.
4. Laser confocal microscopy was used to study the degree of utilization of the remaining oil after heavy oil activator flooding. The results revealed a significant decrease in the saturation level of the clusterlike and membranelike remaining oil systems (from 23.33% to 18.58% and from 14.14% to 10.26%, respectively). Additionally, a fluorescence detection analysis revealed a slow rate of decline in the light/heavy component contents, indicating a high recovery degree of heavy oil components. The recovery degree increased from 3.22 to 9.10%.
5. CT scanning technology was used to characterize different types of microscopic remaining oil quantitatively. The swept volume increased, and the clusterlike remaining oil was effectively utilized following heavy oil activator flooding. This resulted in a decrease in the saturation level of the remaining oil from 24.16 to 18.91%. Additionally, the interfacial tension was reduced, and the activator caused a disassembly and dispersion, decreasing the membranous-like and columnlike remaining oil content from 11.42% and 7.49% to 9.41% and 5.44%, respectively.

## Figures and Tables

**Table 1:** Microdisplacement data for different types of remaining oil.

Displacement Stage	Remaining Oil Saturation Content (%)				
	Clusterlike	Corner-Shaped	Columnar-Like	Dropletlike	Membranous-Like
Water flooding	28.18	6.76	9.43	4.99	9.89
Heavy oil activator flooding	22.60	4.57	6.82	4.63	6.41

**Table 2:** Data on remaining oil saturation in pores at different displacement stages.

Displacement State	Oil Saturation (%)			
	All Pore Intervals	Less Than 10 ms	10–100 ms	Greater Than 100 ms
Saturated oil-bound water state	74.17	64.94	72.41	82.46
Water flooding	61.63	59.58	61.63	63.12
Heavy oil activator flooding	50.22	50.64	51.46	48.79
Subsequent water flooding	45.94	48.16	47.09	43.28

**Table 3:** Data table for the degree of heavy oil recovery at different displacement stages.

Displacement State	Recovery Degree (%)	Absolute Recovery Degree (%)			Relative Recovery Degree (%)		
		Small Pores (<10 ms)	Middle Pores (10~100 ms)	Macropores (>100 ms)	Small Pores (<10 ms)	Middle Pores (10–100 ms)	Macropores (>100 ms)
Water flooding	16.91	1.99	5.01	9.93	8.26	14.89	23.46
Heavy oil activator flooding	32.29	5.31	9.74	17.28	22.02	28.93	40.84
Subsequent water flooding	38.06	6.23	11.77	20.11	25.84	34.97	47.51

**Table 4:** Data obtained for the remaining oil in different displacement stages using the laser confocal microscopy technique.

Displacement State	Recovery Degree (%)	Remaining Oil Saturation Content (%)						
		Bound Remaining Oil			Semibound Remaining Oil		Free Remaining Oil	
		Membranelike	Particle-Adsorbent-Like	Slitlike	Corner-Shaped	Throatlike	Clusterlike	Intergranular-Adsorption-Like
Water flooding	16.81	14.14	3.79	2.14	6.52	4.38	23.33	4.81
Heavy oil activator flooding	34.31	10.26	3.61	1.84	4.75	3.28	18.58	3.62
Subsequent water flooding	39.76	9.38	3.44	1.70	3.76	2.78	17.27	3.48

**Table 5:** Data on light/heavy components recorded at different displacement stages.

Displacement Stage	Remaining Oil Saturation (%)			Recovery Degree (%)			Ratio of Light/Heavy Components
	Core	Heavy Components	Light Components	Core	Heavy Components	Light Components	
Initial	72.86	29.39	43.48	-	-	-	1.48
Water flooding	59.50	27.04	32.46	18.35	3.22	15.13	1.20
Heavy oil activator flooding	48.16	22.75	25.40	33.91	9.10	24.81	1.12
Subsequent water flooding	44.90	21.92	22.98	38.37	10.23	28.14	1.05

**Table 6:** Data obtained from CT scans conducted on the microscopic remaining oil.

Displacement State	Oil Saturation, S_o_ (%)	Remaining Oil Saturation (%)				
		Clusterlike	Multiporous Form	Columnlike	Dropletlike	Membranous-Like
Initial	71.32	29.12	13.25	9.14	6.77	13.03
Water flooding	59.56	24.16	10.63	7.49	5.86	11.42
Heavy oil activator flooding	47.53	18.91	9.04	5.44	4.74	9.41
Subsequent water flooding	43.14	17.90	7.93	4.90	4.16	8.24

**Table 7:** Quantitative characterization and classification standards of microscopic remaining oil.

Type	Typical Figure	Number of Occupied Pore Throats	Shape Factor	Contact Ratio	Euler Number
Clusterlike	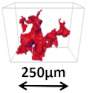	Connected Pore Number > 5	G > 2	C ≥ 0.4	EN ≤ −1
Multiporous form	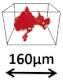	1 < Connected-Pore Number ≤ 5	G > 2	C ≥ 0.4	EN > −1
Columnar-like	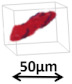	Number of Pore-Throats ≤ 1	G > 2	C ≥ 0.4	EN > 0
Dropletlike	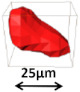	Number of Pore-Throats ≤ 1	G ≤ 2	C = 0	EN > 0
Membranous-like	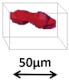	Thickness < 1/3 of pore throat’s diameter	G > 2	C < 0.4	EN > 0

## Data Availability

All relevant data have been presented in this paper.
